# Supplementary material for: Household survey on owned dog population and rabies knowledge in selected municipalities in Bulacan, Philippines: A cross-sectional study
Source: PLoS Negl Trop Dis. 2022 Jan 18;16(1):e0009948. doi: 10.1371/journal.pntd.0009948 (PMC8797173; doi:10.1371/journal.pntd.0009948)
Supplement: S1 Table — (DOCX) [file pntd.0009948.s001.docx]

# Supporting information

# S1 Table. Scoring system

| Question | Answer |  |
| --- | --- | --- |
| Who can get infected with rabies |  | Score (Total=0~6) |
|  | Humans | 2 |
|  | Cats | 1 |
|  | Dogs | 2 |
|  | Livestock (cattle, pig, sheep) | if all correct=1 |
|  | Poultry |  |
|  | Monkey |  |
|  | Bats |  |
|  | Rodents |  |
| What are the signs of rabies in dogs? |  | Score (Total 0~4) |
|  | Fear of Water | Three or more symptoms mentioned=4,   One or two symptoms mentioned=2 |
|  | Aggressiveness |  |
|  | Paralysis |  |
|  | Runs around aimlessly |  |
|  | Change in behavior |  |
|  | Restlessness |  |
|  | Salivating/drooling |  |
|  | Pica (excessive biting of non-nutritious items) |  |
| What are the signs and symptoms of rabies in humans? | | Score (Total 0~4) |
|  | Fear of Water | Three or more symptoms mentioned=4,  One or two symptoms mentioned=2 |
|  | Aggressive |  |
|  | Paralysis |  |
|  | Delirium |  |
|  | Convulsions |  |
|  | Restlessness |  |
| How can a dog get rabies? |  | Score (total 0~5)* |
|  | Bitten by rabid dog | 4 |
|  | Inborn | -1 |
|  | Hot weather | -1 |
|  | Not bathed | -1 |
|  | Scavenging garbage | -1 |
|  | Dirty food | -1 |
|  | Unvaccinated | 1 |
| How can a human get rabies? |  | Score (total 0~4)* |
|  | Bitten by rabid dog | 2 |
|  | Bitten by dog | 1 |
|  | Licked on broken skin | 1 |
|  | Licked on intact skin | -1 |
| How will you prevent dogs from getting infected with rabies? | | Score (total 0~4)* |
|  | Vaccination | 2 |
|  | Don't allow to roam freely | 2 |
|  | Bathe regularly | -1 |
| How frequently should dogs be vaccinated against rabies? | | Score (0~2) |
|  | Every year | 2 |
|  | Other answer or unknown | 0 |
| Overall score knowledge |  | 0~29 |
|  |  |  |
| Perception |  |  |
| If you were to encounter a dog you suspect has rabies, what will you do? | | Score (0~6) |
|  | Capture & observe the dog for 14 days | 2 |
|  | Avoid | 1 |
|  | Report to authorities | 2 |
|  | Bring to a veterinarian | 1 |
| What will you do if you suspect your dog has rabies? | | Score (0~6) |
|  | Capture & observe the dog for 14 days | 2 |
|  | Report to authorities | 2 |
|  | Bring to a veterinarian | 2 |
| Overall score practices |  | 0~12 |
| Overall score |  | 0~41 |
| * if total is <0, point become 0 |  |  |
