# Supplementary material for: Household survey on owned dog population and rabies knowledge in selected municipalities in Bulacan, Philippines: A cross-sectional study
Source: PLoS Negl Trop Dis. 2022 Jan 18;16(1):e0009948. doi: 10.1371/journal.pntd.0009948 (PMC8797173; doi:10.1371/journal.pntd.0009948)
Supplement: S2 Table — (DOCX) [file pntd.0009948.s002.docx]

# Supporting information

# S2 Table. Practices of households with bite victims (*n=*81, 64 households)

| Practice of bite victims (N=81* ,64households) | | N | % |
| --- | --- | --- | --- |
| What was done as first aid to the patient after the last biting incident? | |  |  |
|  | Nothing | 2 | 2.5 |
|  | Washed | 66 | 81.5 |
|  | Washed with water | 17 | 21 |
|  | Washed with soap and water | 49 | 60.5 |
|  | Applied garlic on wound | 4 | 4.9 |
|  | Induced bleeding | 12 | 14.8 |
|  | Applied antiseptic on wound (iodine, betadine) | 1 | 1.2 |
|  | Take antibiotics | 0 | 0 |
| What was done to the patient after administering first aid? | |  |  |
|  | Nothing | 11 | 13.6 |
|  | Consulted traditional healer | 10 | 12.4 |
|  | Consulted at health facilities | 62 | 76.5 |

*n=3 missing data
