# Supplementary material for: Household survey on owned dog population and rabies knowledge in selected municipalities in Bulacan, Philippines: A cross-sectional study
Source: PLoS Negl Trop Dis. 2022 Jan 18;16(1):e0009948. doi: 10.1371/journal.pntd.0009948 (PMC8797173; doi:10.1371/journal.pntd.0009948)
Supplement: S3 Table — (DOCX) [file pntd.0009948.s003.docx]

# Supporting information

# S3 Table. Number of human and animal rabies cases in Bulacan 2015-2018 (Department of Health – Region 3 Office, Department of Agriculture – Region 3 Office)

|  | Human Rabies | | | | | Animal Rabies | | | | |
| --- | --- | --- | --- | --- | --- | --- | --- | --- | --- | --- |
|  | 2015 | 2016 | 2017 | 2018 |  | 2015 | 2016 | 2017 | 2018 |  |
| Region3 | 29 | 33 | 37 | 58 |  | 180 | 156 | 241 | 252 |  |
| Bulacan Province | 5 | 11 | 7 | 9 | Total  32  Median  8 | 44 | 25 | 29 | 36 | Total  134  Median  32.5 |
