# Supplementary material for: Household survey on owned dog population and rabies knowledge in selected municipalities in Bulacan, Philippines: A cross-sectional study
Source: PLoS Negl Trop Dis. 2022 Jan 18;16(1):e0009948. doi: 10.1371/journal.pntd.0009948 (PMC8797173; doi:10.1371/journal.pntd.0009948)
Supplement: Attachment — Submitted filename: Dizon et al peer review.pdf [file pntd.0009948.s005.pdf]

Introduction

Line 62: rabid dogs are not the most significant hazard for rabies in Western and Central Europe, North America, Japan, etc.

Line 65: “rabies free status” usually refers to eradication of rabies in reservoirs species, which is not achieved by administering human PEP.

Line 73: I presume that you mean post-exposure prophylaxis” instead of “treatment of rabies”, as rabies cannot be treated.

Line 73: the link to reference 6 is broken.

Lines 73-76: this is interesting and important, but please provide some more detail about the trend. When was the ABTC program implemented and fully functional with 1milion PEP provided per year?

Lines 80-81: This is an understatement. Dog population estimates are critical to estimate staffing and budget needs, and dog population ecology (i.e. ratio of unowned, owned restrained, owned unrestrained, etc) are critical to decide on the vaccination campaign strategy.

Line 84: please provide a link for reference 12.

Line 93: treatment of dog bites or treatment of rabies?

Line 95: what should dog owners know about the anti-rabies act?

Line 104: incidence of human or dog rabies?

Lines 98-108 (critical) please rephrase the last part of this introduction to clearly state the objectives of this study and the overall methods (cross sectional KAP survey?). It is unclear if this is going to be KAP on dog ownership or on rabies awareness, and whether is includes a dog ecology study. Please also clarify the intended use of the findings (improve education campaigns? Better dog vaccination strategies?). I also suggest you move some of the details on household organisation and land tenure to the methods under “Study site”.

Methods

General comment (critical):

- define “HHD”. What category of dogs does it include (unowned, owned restrained, owned unrestrained, etc)?
- Given that dog population is heterogenous, please provide a bit more information on the study sites in terms of rural vs urban, and other factors that could affect dog population density.

Lines 115-116: the date ranges are nearly identical (2015-2018 and 2014-2018) but the number of cases differs significantly (32 vs 159). Please clarify.

Line 127: Suggest that you specify that the sum of green and red dots represent every household in their respective barangay.

Lines 131-132: please provide details on the survey. Was it a human population survey? Dog ecology survey? Rabies KAP study? Which institution collected the data?

Line 132: Provide additional detail on rational for sample size calculation.

Line 143: was the list of household GPS coordinates randomised?

Line 153: please provide questionnaire as supplementary material.

Line 154: missing word.

Line 169: do you mean the HHD population in each barangay?

Line 171: the survey also thought to determine the total number of humans per barangay? Or are you referring to the survey on line 131? This is unclear.

Line 174: if the objective is to determine vaccination coverage, please add it to the study objective in the introduction and expand on it in the methods. Vaccination data using veterinary records only (as opposed to also asking dog owners or even serology) is likely to severely underestimate vaccination coverage, unless the provincial vet office is truly able to record every dog that receives a rabies vaccine. If that is the case, please provide details.

Line 812: what factors? I assume socio-demographic?

Results

General comments: three decimal point are not needed for such a small study. One decimal point is usual sufficient.

Line 198; IQR is a range; it cannot be a single number.

Line 203: the numbers in brackets are range or IQR?

Line 207: vaccine status is obtained from the survey or from vet office registration?

Line 208: mean dog population per what unit? Household?

Line 209: how is the dog pop per human pop different from the dog:human ratio? One is just the inverse of the other.

Line 211: which survey? The current one or the one on line 131?

Line 213: specify unit (dogs) for numbers in ().

Lines 214-215: this is very important information and goes back to what I said in the introduction: it will be very important to provide information on how dog vaccination occurs and the complexness of dog vaccination records. In particular, what % of vaccinated dogs can be expected to be registered with the Provincial Vet Office?

Line 222: typically, socio-demographic data is presented first in the results.

Line 224: please provide approximate USD equivalent

Line 227: this is not an incidence. Please delete the word.

Line 22: what is the time frame for the bite victims? Cumulative over their lifetime? In the past year? It would be astonishing to have 64 bite victims in 2019 and 84 bite victims over a lifetime.

Line 229: please rephrase. I assume you are meaning that there was a significant association (p=?) between education level and allowing dog to roam freely?

Line 235: please provide the N for each percentage as it is important to understand the denominator (in case respondents did not answer every question).

Line 256: p value is missing for all aORs to determine if significance was met.

Lines 266-269: please rephrase for clarity (split into 2 phrases).

Line 275: how many received PEP? How many were eligible for PEP and did/did not receive it?

Tables 1 & 2: reference them in the text.

Table 2:

- unclear what this p-value compares and what test was used (add this to table footnotes).
- I would modify the table to have the last row as a column so that you can compare biting by socio-demographic status.

Figure 2: were respondents given the choice of "don't know" or refuse to respond? It is also important to provide the number of respondents for each question.

Table 3:

- Place ref variable in the top row for each factor.
- Consider adding a row for dog biting
- Is the OR calculated based on a low score or a high score? From line 266, it seems to be low score.

Discussion

Line 288: awkward phrasing; rather you observed a larger HDD population compared to that when applying the HDR recommended by the national rabies control program.

Line 291: here, and throughout the manuscript, you should make it clear that it is the <u>owned</u> dog:human population ratio.

Line 295: again, please comment and the completeness of provincial vaccination data.

Line 300: what are "random and systemic" methods? Did any study use ecological methods (e.g. sight-resight)?

Lines 305-321: for each study, specify if this the <u>total</u> DHR or the <u>owned</u> DHR. In particular, line 317: is this the <u>total</u> or the <u>unowned</u> DHR?

Line 337: the mapping of every household in your data frame is a strength of the manuscript. Consider emphasizing this.

Line 341-344: move to results.

Line 351-352: ???? This is not necessarily incorrect.

Line 377: possibly, although estimating unowned free roaming dogs is critical for vaccination campaigns and should be done in addition to surveys. I would temper this opinion.
