# Supplementary material for: Household survey on owned dog population and rabies knowledge in selected municipalities in Bulacan, Philippines: A cross-sectional study
Source: PLoS Negl Trop Dis. 2022 Jan 18;16(1):e0009948. doi: 10.1371/journal.pntd.0009948 (PMC8797173; doi:10.1371/journal.pntd.0009948)
Supplement: Attachment — Submitted filename: REVIEW PLOS NTD.pdf [file pntd.0009948.s006.pdf]

PLOS Neglected Tropical Diseases

Identifying gaps in household dog population and rabies knowledge in selected municipalities in Bulacan, Philippines: a cross-sectional study

Manuscript Draft

Manuscript Number: PNTD-D-21-00652

Full Title: Identifying gaps in household dog population and rabies knowledge in selected municipalities in Bulacan, Philippines: a cross-sectional study

Short Title: Household dog population and rabies knowledge in Bulacan

Review Outline

The authors hypothesize that without an accurate estimation of dog population, is not feasible to provide an adequate quantity of animal vaccines, and also that identifying weaknesses in population knowledge about rabies may serve as a focal point for future education campaigns

To address their research topic, they carried out a cross-sectional study in 6 municipalities of Bulacan province in the Philippines, where human rabies cases have been consistently reported. They have obtained the geographic information of all households in the target neighborhoods, followed by a face-to-face survey using a questionnaire, collecting demographic information, data on animal bite incidence, and details on knowledge and practices about rabies. Additional information about dog ownership practices, and estimation of dog population were also included. The authors state that several previous studies attempted to determine the dog: human ratio in the Philippines and found differences between their results and estimation using the 1:10 ratio. They assume the underestimating dog population would lead to insufficient vaccine coverage and cite that the recommendation of the national rabies committee has not been changed because of insufficient data from the dog population survey.

By a household survey in a densely populated area, using household mapping and systematic randomization, they have concluded there might have been a three-fold underestimation of the estimated population, reducing the estimated dog vaccine coverage from around 41% to 15%, far below the recommended 70%. They hypothesize that the results of studies that include both households that own and do not own dogs may more closely represent the actual HHD population, and also argue that complete identification of households or systematic sampling has not been undertaken in any study so far.

They analyzed demographic data and associated areas with insufficient knowledge on rabies to education level and highlighted that residents of households with dogs scored higher than those without dogs, pointing out the lack of

understanding that only dogs with rabies can transmit the disease to humans and other dogs.

Finally, they concluded that the current dog: human ratio, 1:10, results in an underestimated target for dog vaccination and consequently, an inadequate vaccination coverage. Further, they identified weakness and improvement areas in rabies knowledge, particularly on how humans and animals get rabies. They recommend the revision of the dog: human ratio. and have proposed content analysis of the existing rabies health education materials.

Overall impressions

The authors conducted a well elaborate survey, focused upon precise household mapping and systematic sampling method randomized, avoiding selection bias that took place in previous studies.

Although the authors bring the inadequate ratio and the lack of a precise dog population as an impairment to achieve the recommended coverage in dog vaccination, no elements are supporting that filling this gap will improve the coverage. Explanation about the previous dog vaccination campaigns is missing. More information about the difficulties faced during previous campaigns of dog vaccination would allow a more consistent analysis. For example, when the massive animal vaccination happens, Is there a shortage of canine vaccines? Additionally, more information about the occurrence of human rabies in the Philippines (a table with the historical series would be enough) will provide a basic understanding of the local situation.

The main strengths of this paper are that it addresses relevant information for rabies control, identifying gaps in dog population, and rabies knowledge. The authors objectively identified limitations in the community's knowledge of rabies, providing specific targets for educational campaigns and also informing which sources of information were more efficient and those that remained underutilized. Despite the no evidenced conclusion relating the non-achievement of the ideal vaccination coverage to incorrect dog: human ratio, and the rather small temporal and spatial extent of the study, the paper brings valuable information, suggestions and solutions, and it should make, after minor revision, a relevant contribution to policy makers in the field of rabies control in the Philippines.

Suggestions for improvement

Line 28
What is “Last few years”? Five years? Decade?

Line 29
For a good summarizing and quick identification whether the paper meets what readers are looking for, I suggest providing a quick definition (such as neighbourhoods) for "barangays"

Line 30
In what period? The highest in the last decade? Five years?

Line 66
Suggest detailing in “free of canine (dog) rabies”

Line 77

Doesn’t make sense. How does PEP in humans impact rabies in dogs? What is this "little impact"?

Line 80
Carrying out the vaccination without the precise number is a challenge, but it is possible to be done. For example, developing countries in America which also did not have an accurate estimate of their canine population and managed to drastically reduce/eliminate dog-related rabies. Suggest rephrase

Line 87
The authors cite several studies about dog population, but the recommendation of the national rabies committee has not been changed because of insufficient data from the dog population survey. Is there a reference from the national rabies committee citing the insufficient data? The previous studies were considered by the national rabies committee?

Line 115
This phrase is not clear. How many cases? 32 cases and also 159 cases in the same province? These 159 (2014 to 18) comprise the 32? The electronic site, cite as reference, with the manual of the Philippines National Rabies Prevention and Control Program it is not working

Line 131
What is Northern Samar? A Province?

Line 157
Were those who had never heard of rabies also counted to the knowledge and perception of the respondents regarding rabies?

Line 213
Is 2018 the only data available? Is there a trend in dog vaccination coverage over the years? How is it?

Line 224
I suggest to convert to US$ to facilitate the understanding

Line 323
Even with the current ratio, the coverage is far below. What are the difficulties alleged?

Line 393
There is a correlation, but there is no causation (consequence). I suggest rephrase
