# Supplementary material for: Household survey on owned dog population and rabies knowledge in selected municipalities in Bulacan, Philippines: A cross-sectional study
Source: PLoS Negl Trop Dis. 2022 Jan 18;16(1):e0009948. doi: 10.1371/journal.pntd.0009948 (PMC8797173; doi:10.1371/journal.pntd.0009948)
Supplement: Attachment — Submitted filename: Response to reviewer 2 - 20210914.docx [file pntd.0009948.s007.docx]

**Response to reviewer #2**

**Title:**

Household survey on owned dog population and rabies knowledge in selected municipalities in Bulacan, Philippines: a cross-sectional study

**Manuscript Number:**

PNTD-D-21-00652

**Reviewer #2**

We appreciate all the valuable comments provided by the reviewer. Following the reviewer’s comments, we have revised the manuscript and have indicated the line location of the revisions in bold.

### Comment 1

Line 28

What is “Last few years”? Five years? Decade?

### Response to comment 1

We have revised the statement to identify the time period as decade. Later in the text, we also specified the inclusive years (2008-2018) and added an average and range values. **(Line 23)**

### Comment 2

Line 29

For a good summarizing and quick identification whether the paper meets what readers are looking for, I suggest providing a quick definition (such as neighborhoods) for "barangays"

### Response to comment 2

We have used the term communities for the abstract but used barangays later in text and provided a quick definition. **(Line 28, 136-137)**

### Comment 2

Line 30

In what period? The highest in the last decade? Five years?

### Response to comment 2

The region had the highest number in 2018. We have added the number of human and animal rabies cases in Bulacan for the period of 2015-2018 as supporting information (S3 Table).

### Comment 3

Line 66

Suggest detailing in “free of canine (dog) rabies”

### Response to comment 3

We have revised this section extensively and we have removed this statement for clarity.

### Comment 4

Line 77

Doesn’t make sense. How does PEP in humans impact rabies in dogs? What is this "little impact"?

### Response to comment 4

This is an error on our part. We have revised this section extensively and we have removed this statement for clarity.

### Comment 5

Line 80

Carrying out the vaccination without the precise number is a challenge, but it is possible to be done. For example, developing countries in America which also did not have an accurate estimate of their canine population and managed to drastically reduce/eliminate dog-related rabies. Suggest rephrase

### Response to comment 5

We have rephrased this statement and identified key points how this information would be essential. **(Line 88-90)**

### Comment 6

Line 87

The authors cite several studies about dog population, but the recommendation of the national rabies committee has not been changed because of insufficient data from the dog population survey. Is there a reference from the national rabies committee citing the insufficient data? The previous studies were considered by the national rabies committee?

### Response to comment 6

Upon consulting the manual of procedures, there were no cited reason for not changing the data. There was no indication in the manual of procedures that they are aware of the previous studies and they were not cited.

### Comment 7

Line 115

This phrase is not clear. How many cases? 32 cases and also 159 cases in the same province? These 159 (2014 to 18) comprise the 32?

### Response to comment 7

This is an error on our part. We have meant to specify the figures for human and animal rabies cases but failed to identify which on the text. **(Line 133-135)**

### Comment 8

The electronic site, cite as reference, with the manual of the Philippines National Rabies Prevention and Control Program it is not working

### Response to comment 8

We have now fixed the hyperlink and they should now direct to the manual of procedures of the National Rabies Prevention and Control program.

### Comment 9

Line 131

What is Northern Samar? A Province?

### Response to comment 9

Yes, Norther Samar is a province in the Philippines. We have corrected this in text.

### Comment 10

Line 157

Were those who had never heard of rabies also counted to the knowledge and perception of the respondents regarding rabies?

### Response to comment 10

Yes, they were still ask various things about rabies.

### Comment 11

Line 213

Is 2018 the only data available? Is there a trend in dog vaccination coverage over the years? How is it?

### Response to comment 11

Our vaccine coverage estimate is based on the data given by the provincial veterinary office. This data is limited to the government led animal rabies vaccination efforts and does not include vaccination outside this program. It is expected that by using this information, vaccine coverage would be underestimated. However, this is the only available data at the time of study and provides a partial measure of vaccine coverage

### Comment 12

Line 224

I suggest to convert to US$ to facilitate the understanding

### Response to comment 12

We have added US$ to the income figures.

### Comment 13

Line 323

Even with the current ratio, the coverage is far below. What are the difficulties alleged?

### Response to comment 13

We were unable to elicit information regarding the difficulties in animal vaccine coverage as it was not part of the study objectives. However we added an older reference that enumerates some of the difficulties encountered during a vaccination campaign. **(Line 345-348)**

### Comment 14

Line 393

There is a correlation, but there is no causation (consequence). I suggest rephrase

### Response to comment 14

We have revised the statement based on this comment. **(Line 449-451)**
